# Evaluating the efficacy of Seattle-PAP for the respiratory support of premature neonates: study protocol for a randomized controlled trial

**DOI:** 10.1186/s13063-018-3166-6

**Published:** 2019-01-18

**Authors:** Carl H. Backes, Jennifer L. Notestine, Jane M. Lamp, Jeanne C. Balough, Allison M. Notestine, Crystal M. Alfred, Juli M. Kern, Michael R. Stenger, Brian K. Rivera, Mohannad Moallem, Randy R. Miller, Apurwa Naik, Jennifer N. Cooper, Christopher R. Howard, Stephen E. Welty, Noah H. Hillman, John A. F. Zupancic, Larissa I. Stanberry, Thomas N. Hansen, Charles V. Smith

**Affiliations:** 1 Center for Perinatal Research, The Research Institute at Nationwide Children’s Hospital, 700 Children’s Drive, Columbus, OH 43205 USA; 2 Department of Pediatrics, The Ohio State University Wexner Medical Center, Columbus, OH USA; 3 The Heart Center, Nationwide Children’s Hospital, Columbus, OH USA; 4 OhioHealth Research Institute, Riverside Methodist Hospital, Columbus, OH USA; 5 Pediatrix Medical Group of Ohio, Columbus, OH USA; 6 Mt. Carmel St. Ann’s Hospital, Westerville, OH USA; 7 OhioHealth, Grant Medical Center, Columbus, OH USA; 8 Center for Surgical Outcomes, The Research at Nationwide Children’s Hospital, Columbus, OH USA; 9 Division of Epidemiology, The Ohio State University College of Public Health, Columbus, OH USA; 10 Seattle Children’s Research Institute, Seattle Children’s Hospital, Seattle, WA USA; 11 Seattle Children’s Neonatology Program, CHI Franciscan Health, Tacoma, WA USA; 12 SSM Health, Cardinal Glennon Children’s Hospital, St. Louis University, St. Louis, MO USA; 13 Division of Newborn Medicine, Harvard Medical School, Boston, MA USA; 14 Department of Neonatology, Beth Israel Deaconess Medical Center, Boston, MA USA; 15 Minneapolis Heart Institute Foundation, Minneapolis, MN USA

**Keywords:** Neonate, Continuous positive airway pressure (CPAP), Preterm Infant, Infant, Costs, Costs analysis, Randomized controlled trial

## Abstract

**Background:**

At birth, the majority of neonates born at <30 weeks of gestation require respiratory support to facilitate transition and ensure adequate gas exchange. Although the optimal approach to the initial respiratory management is uncertain, the American Academy of Pediatrics endorses noninvasive respiratory support with nasal continuous positive airway pressure (nCPAP) for premature neonates with respiratory insufficiency. Despite evidence for its use, nCPAP failure, requiring intubation and mechanical ventilation, is common. Recently, investigators have described a novel method to deliver bubble nCPAP, termed Seattle-PAP. While preclinical and pilot studies are encouraging regarding the potential value of Seattle-PAP, a large trial is needed to compare Seattle-PAP directly with the current standard of care for bubble nCPAP (Fisher & Paykel CPAP or FP-CPAP).

**Methods/design:**

We designed a multicenter, non-blinded, randomized controlled trial that will enroll 230 premature infants (22^0/7^ to 29^6/7^ weeks of gestation). Infants will be randomized to receive Seattle-PAP or FP-CPAP. The primary outcome is respiratory failure requiring intubation and mechanical ventilation. Secondary outcomes include measures of short- and long-term respiratory morbidity and cost-effectiveness.

**Discussion:**

This trial will assess whether Seattle-PAP is more efficacious and cost-effective than FP-CPAP in real-world practice among premature neonates.

**Trial registration:**

ClinicalTrials.gov, NCT03085329. Registered on 21 March 2017.

**Electronic supplementary material:**

The online version of this article (10.1186/s13063-018-3166-6) contains supplementary material, which is available to authorized users.

## Background

Neonates face significant challenges in the successful transition from fetal life [1, 2]. Aeration of liquid-filled lungs and the establishment of functional residual capacity are critical tasks in a newborn’s adaptation to the extrauterine environment. Beginning with their initial breath after birth, term infants clear fluid from their lungs and establish functional residual capacity to facilitate gas exchange [2]. In contrast, among many preterm neonates, surfactant deficiency, immature epithelial sodium channels, instability of the thorax, respiratory fatigue, and apnea contribute to respiratory insufficiency, often resulting in respiratory failure and the need for respiratory support [3–5]. Although the best approach to the initial respiratory management of these infants is uncertain, evidence that even brief exposures to tracheal intubation and mechanical ventilation cause lung injury have led to interest among health-care providers in the use of noninvasive respiratory support devices, primarily nasal continuous positive airway pressure (nCPAP) [6].

The American Academy of Pediatrics has endorsed the use of nCPAP among premature neonates with respiratory distress [7]. This recommendation is based on data indicating lower rates of bronchopulmonary dysplasia (BPD) in infants treated with nCPAP than are observed with more invasive modes of ventilatory support [8–10]. However, nCPAP failure, requiring intubation and mechanical ventilation in preterm infants, is common, with failure rates exceeding 50% in large clinical trials [8–10]. In developed countries, CPAP failure is associated with greater morbidity; in developing countries, CPAP failure is associated with greater mortality [11, 12].

More recently, bubble nCPAP has re-emerged as a potential strategy to address high nCPAP failure rates [13]. Although there are limited data, neonates on bubble nCPAP had a lower incidence of respiratory failure (tracheal intubation and mechanical ventilation) than did infants supported on ventilator-derived CPAP [12, 14]. Despite evidence that bubble nCPAP may be advantageous for preterm infants, the optimal delivery for newborns with respiratory distress is unknown. To address high failure rates associated with CPAP among preterm neonates, investigations of novel strategies to deliver more effective bubble nCPAP are warranted [15]. One potential strategy is the use of Seattle-PAP [12, 13, 16, 17].

**Fig. 1:**
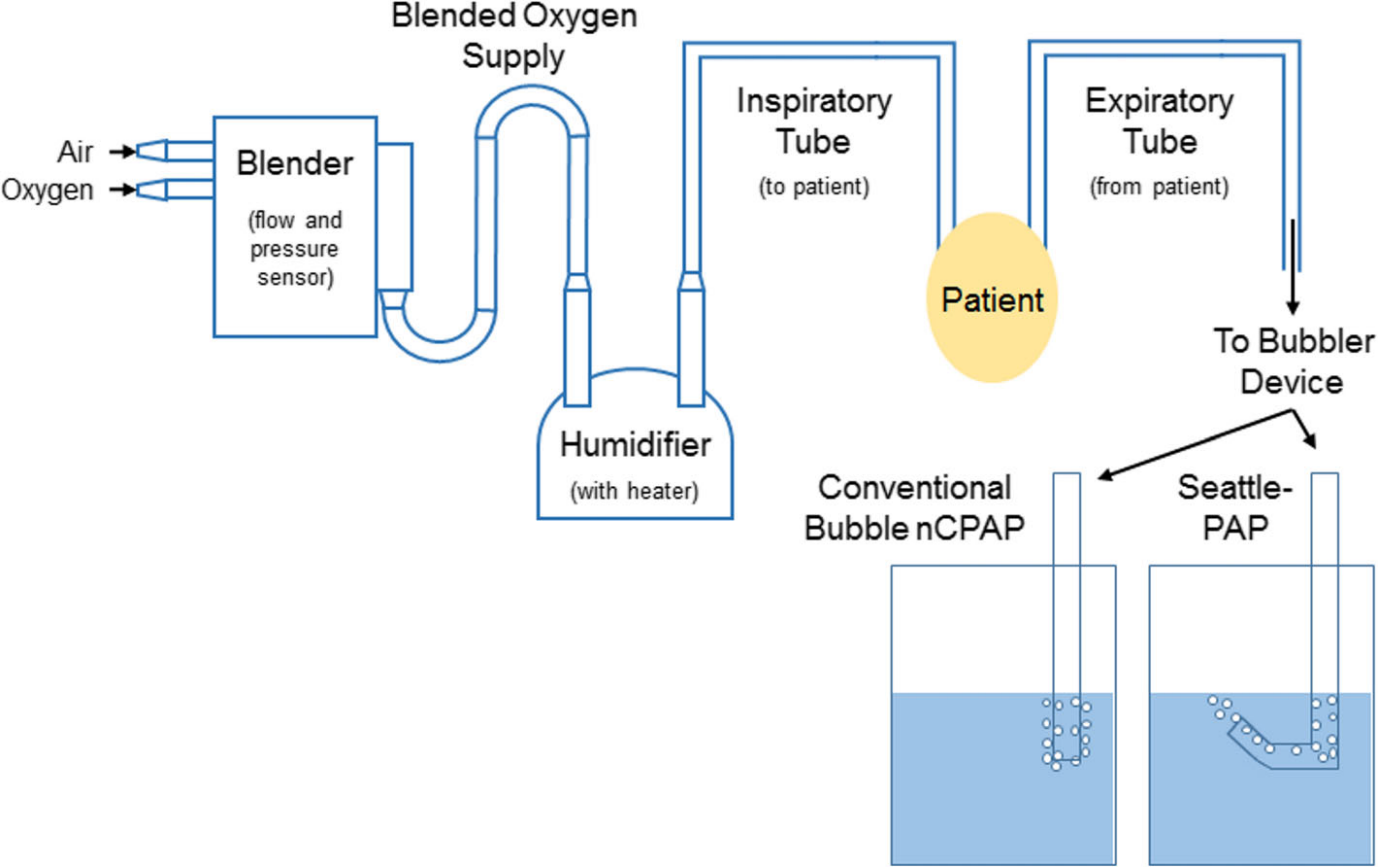
Schematic showing the primary components of a bubble nCPAP circuit. The differences in conventional and Seattle-PAP bubbler devices are shown. All other elements of the CPAP circuit are identical between the groups

In general, bubble nCPAP delivery systems consist of six primary components (Fig. 1): (1) sources of air, oxygen, and other breathing gases, (2) a blender to mix these gases together, (3) a gas heater and humidifier, (4) inspiratory and expiratory tubing (breathing circuit), (5) a patient interface (e.g., bi-nasal prongs or a nasal mask), and (6) a pressure generator (e.g., bubbler device). The design of the bubbler device can take many forms, but they generally comprise a tube with its distal end submerged in a body of water. The airway pressure provided by the system is determined largely by the depth of the distal end of the bubbler tube below the surface of the water (5 cm below surface = 5 cm H_2_O). In conventional bubble nCPAP systems, the bubbler tube points straight down (perpendicular to the surface of the water), which we define as 0°. With Seattle-PAP, the vertical section of the bubbler tube is connected to a horizontal section, which is connected to an upward sloping section, which we optimized at 135°.

Preclinical evidence demonstrated that this 135° modification provides fluctuations in airway pressures, including lower-frequency pressure oscillations than those created by conventional systems [17, 18]. Moreover, a recent study showed that prematurely born neonates (mean gestational age [GA] of 29 weeks) with minimal parenchymal lung disease exerted less effort in spontaneous breathing with a Seattle-PAP than a conventional bubble nCPAP (Fisher & Paykel CPAP or FP-CPAP) [13]. While the results of preliminary studies are encouraging, safety and efficacy data among smaller, more immature preterm infants are lacking.

If the Seattle-PAP arm proves superior to conventional bubble nCPAP, the likely benefits would be large, with clear applications in both the developed world and in low- and middle-income countries. However, to recommend Seattle-PAP over the current standard of care for bubble nCPAP (FP-CPAP), a large comparative trial is needed. Additionally, since respiratory failure is associated with higher daily costs related to more intensive monitoring and personnel requirements [19], Seattle-PAP may result in lower overall treatment costs if it prevents respiratory failure. Similarly, since infants failing CPAP stay in hospital longer than those successfully supported on CPAP, the total costs should be lower if use of Seattle-PAP is associated with a lower incidence of CPAP failure than FP-CPAP [20]. To estimate the cost-effectiveness of Seattle-PAP, a formal economic evaluation ancillary to the proposed randomized controlled trial (RCT) is necessary [21].

The present report describes the rationale and design for an ongoing RCT that aims to compare the effectiveness of Seattle-PAP versus conventional FP-CPAP in the prevention of respiratory failure of premature neonates born at 22^0/7^–29^6/7^ weeks’ GA. This study protocol was written in accordance with the Standard Protocol Items: Recommendations for Interventional Trials (SPIRIT) guidelines. The SPIRIT Checklist has been included in Additional file 1.

### Hypothesis

We are testing the hypothesis that premature neonates supported by Seattle-PAP will have lower rates of respiratory failure (need for tracheal intubation and mechanical ventilation) than will neonates supported with conventional bubble CPAP (FP-CPAP).

### Aims

The primary objective of this study is to compare the rates of respiratory failure from 72 h post-delivery to 32 weeks’ postmenstrual age in neonates born at 22^0/7^ to 29^6/7^ weeks’ GA who receive either Seattle-PAP or conventional bubble nCPAP (FP-CPAP).

## Methods/design

### Study settings

This is a multicenter non-blinded RCT in premature neonates born at 22^0/7^ to 29^6/7^ weeks’ GA. The trial is ongoing at five hospitals within the Neonatal Research Network (NRN) of the Nationwide Children’s Hospital (NCH): NCH Main Campus, Ohio State University Wexner Medical Center, OhioHealth Riverside Methodist Hospital, Mt. Carmel St. Ann’s Hospital, and OhioHealth Grant Medical Center. NCH is a Level IV referral facility covering Central Ohio and parts of West Virginia and Kentucky. All neonatal intensive care units (NICUs) in NCH-NRN routinely care for premature neonates with respiratory insufficiency, using FP-CPAP as a non-invasive respiratory support modality. Participating sites did not use Seattle-PAP prior to study commencement.

All participating sites administer an exogenous surfactant if the infant requires tracheal intubation for respiratory distress. One NICU (Mt. Carmel St. Ann’s Hospital) has some experience with less invasive surfactant administration, in which infants on bubble nCPAP are given a surfactant via thin catheters directly placed into the trachea while they are breathing spontaneously, thereby avoiding the need for intubation for surfactant administration [22]. All participating centers have 24-h on-site neonatal staff.

The Institutional Review Board (IRB) of NCH approved the trial (16–00678). The IRB at Mt. Carmel St. Ann’s Hospital granted separate approval for the trial (170817–2). Of note, prior to study commencement, the Seattle-PAP device received clearance from the U.S. Food and Drug Administration via the 510(k) pathway (K131502; 11 October 2013).

### Inclusion and exclusion criteria

The inclusion criteria are as follows:

1. parental consent and/or legal guardian consent given to participate in this research study
2. preterm infants delivered at 22 to 30 weeks of completed gestation by best obstetrical estimate
3. weight <1500 g at birth
4. able to be weaned from mechanical ventilator support within 72 h of birth

The exclusion criteria are as follows:

1. Infants with known major congenital cardiac (e.g., transposition of the great arteries), pulmonary (e.g., pulmonary and/or tracheal hypoplasia), or physiological (e.g., anencephaly, omphalocele, congenital diaphragmatic hernia) anomalies. For the purposes of this definition, common preterm cardiac issues such as patent ductus arteriosus and patent foramen ovale or atrial septal defect will not be grounds for exclusion.
2. Infants with known genetic anomalies (e.g., trisomy 21).
3. Infants born to mothers who are unable to give informed consent.

#### Recruitment

**Fig. 2:**
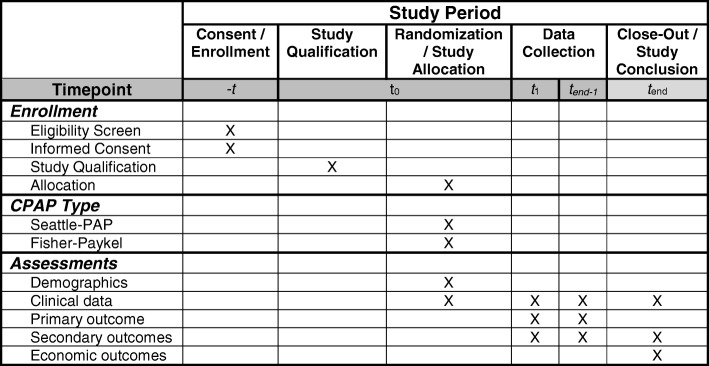
Schedule of enrollment, interventions, and assessments during the study

A member of the study team will approach parents of potentially eligible infants with threatened preterm delivery between 22^0/7^ and 29^6/7^ weeks’ GA to offer study participation and to obtain written informed consent. Consent will be obtained by team members who have been trained in obtaining consent for clinical trials and who are familiar with the trial protocol. Whenever possible, consent will be obtained by someone not directly involved in the clinical care of the infant. If it is not possible to obtain consent during the antenatal period, then as soon as possible following the birth, a member of the study team will approach the infant’s parents or legal guardians to obtain consent (up to 72 h post-delivery). Parents or guardians of infants who are not yet eligible but are likely to become eligible (e.g., infants requiring mechanical ventilation who are likely to transition from mechanical ventilation to bubble nCPAP prior to 72 h) will also be approached. Fig. 2 is the schedule of enrollment, interventions, and assessments. Recruitment began in March 2017.

#### Randomization

**Fig. 3:**
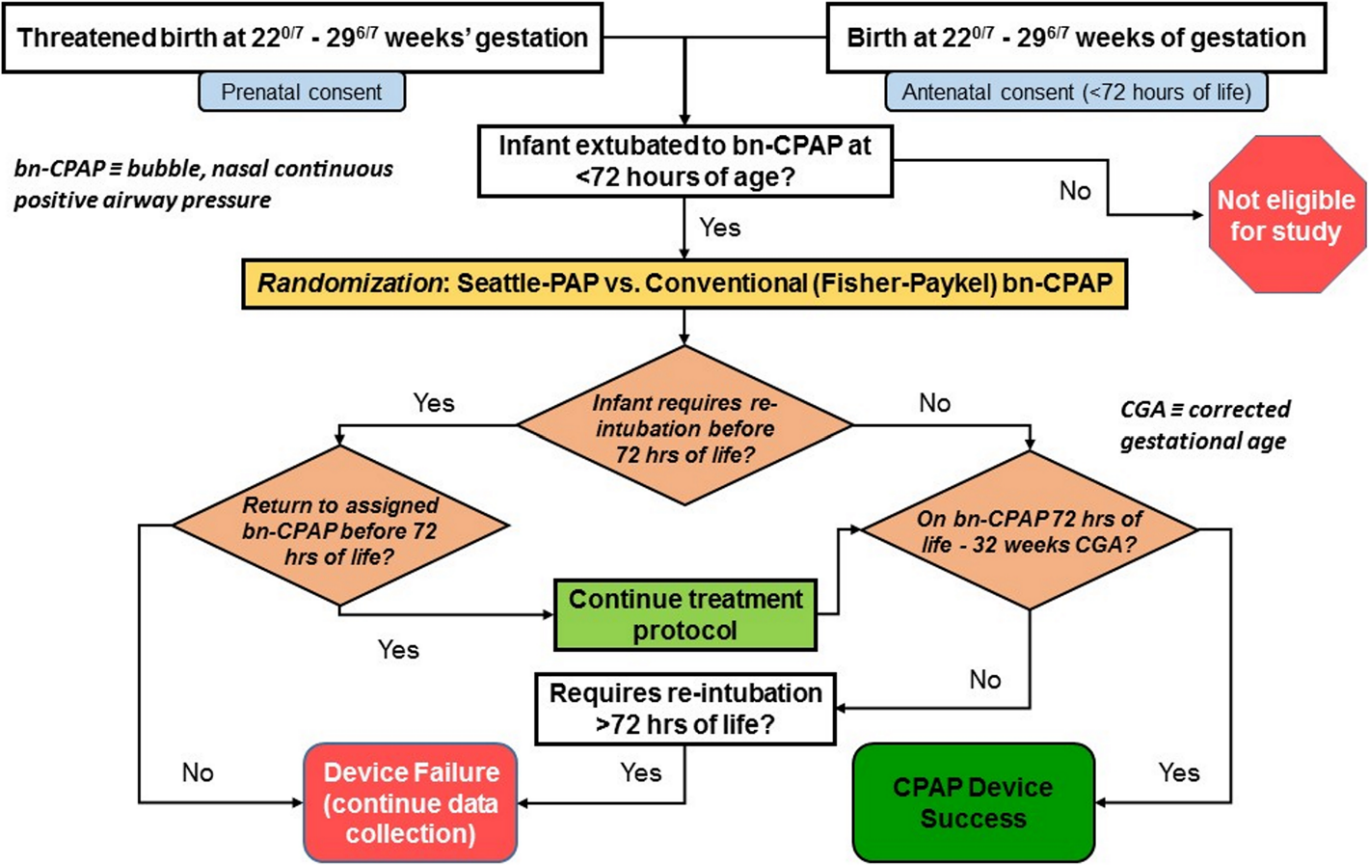
Study flow chart. bn-CPAP bubble nasal continuous positive airway pressure, CGA corrected gestational age

Enrolled eligible infants are randomly assigned to one of two treatment arms (Seattle-PAP vs. FP-CPAP) (Fig. 3). The allocation sequence is generated using an online, computer-generated randomizer (https://sealedenvelope.com), with a block size of 6, stratified by GA (27–29^6/7^ and 22–27 weeks’ GA). We did not stratify by site, as the treatment of premature neonates is based on shared guidelines throughout the NCH-NRN [23]. Multiple gestations (twins or triplets) are assigned to the same treatment arm.

#### Intervention

The allocated treatment is applied immediately after randomization. Infants whose condition cannot be maintained with the assigned method of noninvasive respiratory support will be intubated and the originally assigned intervention resumed after extubation. In the NCH-NRN, bubble nCPAP is given through nasal prongs or a mask with an initial pressure of 5 to 6 cm H_2_O. As described above, the primary difference between the groups is the bubbler, which generates the pressure for the circuit (Fig. 1).

#### Blinding

Blinding of the allocated treatment is not feasible, as the mode of respiratory support is apparent to health-care professionals and families. However, statisticians performing the data analyses will be blinded to group assignment. All enrolled neonates will be given clinical care according to established guidelines, including weaning, extubation, and reintubation [23]. Moreover, the primary outcome (respiratory failure, as defined below) is based on an objective set of criteria applied to both groups.

#### Clinical and respiratory guidelines

Because the duration and type of respiratory support are critical end points, we have taken a number of steps to ensure that respiratory support is applied similarly to both groups: (1) NCH-NRN follows a respiratory algorithm for care (see Additional file 2: Figure S1) and (2) the thresholds for achieving the primary end point of respiratory failure are clearly defined (see below). In addition to standardized respiratory support, all aspects of neonatal management and treatment will be in accordance with local guidelines [23]. Internal audits to investigate maintenance and adherence to the guidelines are conducted on a routine basis.

*Treatment failure* is defined as any of the following:

1. Tracheal intubation within 72 h for surfactant administration after initiation of bubble nCPAP and then not extubated by 72 h.
2. Tracheal intubation of the infant or support with biphasic CPAP (SiPAP) in the NICU after 72 h and up to 32 weeks’ GA. As adjudicated by an independent party, tracheal intubation for non-respiratory issues after 72 h and up to 32 weeks’ GA (e.g., surgery for retinopathy of prematurity) will not be considered a treatment failure.
3. The infant cannot sustain SpO_2_ of at least 90%, despite noninvasive respiratory support of up to and including 8 cm H_2_O bubble nCPAP, and FiO_2_ greater than 0.40 for more than 1 h.

#### Study conclusion

Patient participation in the study will conclude at 36 weeks’ corrected GA or at discharge, whichever comes first.

#### Outcomes

The primary outcome is treatment failure (as defined above). We will capture important secondary outcomes, including short- and longer-term respiratory morbidities (Additional file 3: Table S1).

With a birth to hospital discharge time horizon, a cost-effectiveness analysis from the perspective of the health-care system was planned a priori and will be conducted alongside the RCT. From a health-care sector perspective, cost-effectiveness analyses include formal health sector (medical) costs, such as those paid by a third party such as government or a private insurer [24].

#### Sample size

Using data from other recent large neonatal randomized trials with similar populations, including the SUPPORT trial and the COIN trial [9, 10], as well as contemporary data within the NCH-NRN, we estimate that the proportion of patients experiencing respiratory failure in the control arm (FP-CPAP) will be 50%. Based on preclinical work, we estimate the failure rate in the Seattle-PAP arm will be 30% [13, 17]. Accounting for two interim analyses, 200 neonates are needed with a two-tailed type I error rate of 0.05 and a power of 80% [25]. Multiples will be randomized as a set to the same study arm, requiring an inflation of the estimate by 1.12 to allow for the design effect due to clustering [26, 27]. Thus, the calculated sample size is 115 infants per treatment arm.

#### Data collection

Except for data related to the screening log, all remaining clinical data will be obtained from the electronic medical records (Additional file 4: Table S2).

Routinely available costs of inpatient stay will be sourced from the hospital costing units. Direct medical resource utilization will be ascertained through collection of itemized billing records and UB-04 forms, a uniform billing statement recommended by the National Uniform Billing Committee and utilized for reporting of hospital expenditures by third-party payers including the Centers for Medicare and Medicaid Services (CMS). We will convert hospital-reported charges to costs by applying the appropriate CMS cost center specific ratio of costs to charges. Total hospital costs will be the sum of the product of the number of days in each cost category and the calculated per diem costs. Physician professional fees for the initial hospitalization will be based on CMS reimbursement levels for each day of stay and non-bundled procedures.

### Safety

#### Data and safety monitoring board

We have assembled a data and safety monitoring board (DSMB) to protect study subjects and monitor the overall conduct of the trial. Prior to study commencement, the four-person DSMB agreed to the following activities: (1) review the protocol and amendments; (2) participate in the development, finalization, and approval of the DSMB charter; (3) recommend discontinuation of the trial for safety reasons; (4) recommend changes to the study protocol (amendments) for safety reasons; (5) evaluate emergent safety information, evaluate any risk, and identify any potential safety concerns for study patients; (6) request additional data not included in reports, if deemed necessary for effective safety monitoring; (7) communicate DSMB findings and recommendations (to stop, continue, or modify the study) to the site principal investigator (PI; CHB); and (8) provide written minutes following scheduled and ad hoc meetings.

Adverse events and their relationship to study, severity, time of experience, expectation, actions taken to resolve the event, and final outcome will be recorded. All serious adverse events (SAEs) will be sent within 48 h to the DSMB and local IRB. A SAE for this study is any untoward medical occurrence that is believed by the investigators to be causally related to the study intervention and results in any of the following: a life-threatening condition (that is, immediate risk of death), severe or permanent disability, or prolonged hospitalization. SAEs occurring after a subject is discontinued from the study will not be reported, unless the investigators feel that the event may have been caused by the study device. All SAEs will be followed until a satisfactory resolution is achieved or until the health-care provider responsible for the care of the participant deems the event to be chronic or the patient to be stable. All expected and unexpected SAEs, whether or not they are attributable to the study intervention, will be reviewed by the site PI (CHB) to determine if there is a reasonable suspected causal relationship to the intervention. If the relationship is reasonable, SAEs will be reported to DSMB and local IRB for consideration.

#### Interim analysis

Interim analyses were conducted by the DSMB following the enrollment of 25 and 110 infants, respectively. The analyses compared the two groups with respect to efficacy, safety, and futility. A Haybittle–Peto stopping guideline was set at *P* < 0.001 for each interim analysis. The interim analyses were completed in June 2017 and January 2018. Based on these analyses, and on safety reviews conducted to date, the DSMB recommended that the trial continue without modification.

#### Duration of study

The projected duration of the study is 3 years, including 2½ years for subject recruitment.

#### Training of clinical staff

Comprehensive education and training were undertaken to ensure technical proficiency and compliance with the intervention and study protocol at all sites. The site PI (CHB), study coordinator (JLN), and nursing leadership on the study (JML and JCB) ran a boot camp at the start of the study for all participating sites, with mandatory attendance for physicians, respiratory therapists, and nursing staff to accomplish the following: (1) review differences between the Seattle-PAP and the conventional FP-CPAP devices; (2) identify areas for device troubleshooting; (3) demarcate study goals and objectives; and (4) review safety protocols, procedures, and guidelines for clinical and respiratory care. This effort was intended to optimize reproducibility and consistency. All centers received detailed written instruction on study protocol. The PI and study coordinator were available 24 h each day to answer any questions or concerns.

#### Data management, processing, monitoring, and security

The data generated in this study will be appropriately documented and checked for validity and accuracy. The data will be entered into a database, then the data will be matched and checked for validity and accuracy by a second person before being endorsed for analysis. A record of all discrepancies and resolutions will be kept by the study coordinator (JLN). Outlier data will be investigated. Data will be primarily managed using REDCap (Research Electronic Data Capture) [28]. REDCap is a secure web-based application designed to support data capture in clinical studies. All data with identifiers will be stored on firewall-protected secure servers. Study monitoring visits are performed by the sponsor and their representatives after the enrollment of 50, 100, 150, and 200 infants, and upon the closure of the study.

### Statistical analysis

#### Clinical outcomes

Analyses will be performed using Statistical Analysis Software (SAS) Enterprise Guide version 7.15 (SAS Institute Inc., Cary, NC, USA) using an as randomized (intention-to-treat) principle to compare the primary outcome between treatment arms. Continuous data will be expressed as means with standard deviations or as medians with ranges, whereas categorical variables will be expressed with frequencies and proportions. The primary outcome, and other binary outcomes occurring in at least 5% of patients, will be analyzed using Pearson chi-squared tests. Less common binary outcomes will be compared using Fisher’s exact tests. Risk differences and the number needed to treat, along with their 95% Newcombe hybrid score confidence intervals will be calculated, and risk ratios, and their 95% Koopman asymptotic score confidence intervals, will also be calculated. Normally distributed, continuous outcomes will be compared using Student’s *t*-test, whereas the Wilcoxon rank-sum test will be used to compare continuous outcomes with skewed distributions.

In additional analyses, log binomial regression models will be used to evaluate heterogeneity in the effect of treatment with Seattle-PAP on the primary outcome. Treatment effect heterogeneity will be explored across several clinical characteristics that are known to be associated with CPAP treatment failure: GA, birth weight, and exposure to antenatal corticosteroids [15]. Treatment effect heterogeneity will be tested by evaluating the significance of interactions between the factors of interest and treatment arm in log binomial regression models that include the factor, treatment arm, and their interaction. Effect estimates within subgroups defined by these factors will be expressed as risk ratios with maximum likelihood-based 95% confidence limits. Regression models will be estimated using generalized estimating equations to account for the inherent correlation expected with multiples [27].

#### Economic outcomes

To inform whether it is cost-effective to incorporate FP-CPAP or Seattle-PAP into the existing health system, a decision analysis will be constructed based on the primary outcome and associated hospital costs. Univariate and probabilistic sensitivity analyses will be conducted to test the impact of uncertainty in the data. We will first compare the mean patient-level costs for each treatment arm, without consideration of effectiveness. Because of the anticipated skewed nature of the cost data, we will model the logarithm of the mean costs directly, using a generalized linear model with a logarithmic link function and gamma distribution [24, 29]. In addition to treatment assignment, covariates will be entered into this model to account for any differences in baseline prognostic indicators (e.g., exposure to antenatal corticosteroids) that are evident despite randomization. The model will take into account clustering among twins.

We will then determine the simultaneous outcome of cost and effect, or value for money, expressed as the incremental cost-effectiveness ratio, which is calculated as the difference in mean cost per patient in the Seattle-PAP and FP-CPAP groups divided by the difference in the mean effect between the two groups. We will determine the statistical uncertainty in the joint distribution of costs and effects using nonparametric bootstrapping, in which we will draw 1000 samples of 220 infants, with replacement, from the study data set [30, 31]. For each of the 1000 samples, we will calculate the mean cost, mean effect, and incremental cost-effectiveness ratio. The results of this analysis will be presented using an incremental cost-effectiveness plot and cost-effectiveness acceptability curves [32]. Parameter uncertainty, for variables such as price weights, will be estimated using a deterministic sensitivity analysis, in which the results are generated again after varying the parameter through its plausible range [30].

### Dissemination of results

The results of the trial will be published in a peer-reviewed journal with open access. To facilitate sharing of the clinical research data, the data from the present study will be made available at the global data sharing enterprise Vivli (https://vivli.org/resources). The Vivli platform provides an independent data repository, search engine, and a secure cloud-based analytics platform.

### Study progress

The trial began recruiting in March 2017. It is expected that recruitment for the study will be completed in 2019.

## Discussion

Previous investigators have shown that tracheal intubation and mechanical ventilation are associated with ventilator-induced lung injury and airway inflammation, which contribute to long-term adverse respiratory consequences [33, 34]. Walsh et al. reported that the duration of tracheal intubation and mechanical ventilation among premature neonates is associated with an increased risk of death or survival with neurocognitive impairment [35]. These observations have led health-care providers to pursue less invasive forms of respiratory support for premature neonates, including nCPAP [9, 10].

nCPAP is an established alternative to intubation and mechanical ventilation for premature infants with respiratory distress [7]. Although meta-analytic data show that early adoption of nCPAP was associated with lower risk of BPD than was observed with intubation and ventilation, a high proportion of neonates are not adequately supported on nCPAP and require tracheal intubation and mechanical ventilation (CPAP failure) [8–10]. In fact, the data suggest that 34 to 83% of premature neonates maintained on nCPAP in the delivery room require tracheal intubation and mechanical ventilation [8–10].

More recently, bubble nCPAP has re-emerged as a potential strategy to address high nCPAP failure rates [13]. Although neonates on bubble nCPAP had lower incidence of respiratory failure (tracheal intubation and mechanical ventilation) than did infants supported on ventilator-derived CPAP, the optimal delivery of nCPAP to premature neonates remains unknown [12, 14, 15]. Recognizing the need for a more efficient bubble nCPAP system among preterm infants [15], investigators designed and developed an alternative device, termed Seattle-PAP [12, 13, 17, 18]. In preclinical studies with juvenile rabbits lavaged to produce pulmonary surfactant deficiency [12, 13, 17, 18], Seattle-PAP provides oscillations in airway pressures at lower frequencies than are observed with other devices [17, 18]. The frequencies of airway pressure oscillations generated by Seattle-PAP are lower than those generated by conventional bubble nCPAP [17, 18]. While conventional bubble CPAP provides stabilization and distension of small airways and alveolar spaces, the range of fluctuations in airway pressure provided by Seattle-PAP may improve recruitment of and ventilation to the low ventilation-perfusion compartments of the lung that contribute to hypoxemia [12].

These observations led to a recent study that compared Seattle-PAP and conventional bubble nCPAP (FP-CPAP) among premature infants with an average GA of 29 weeks. The authors observed, among 40 neonates, reduced breathing effort with Seattle-PAP than with FP-CPAP; however, the study was not designed to detect important clinical differences between the two devices [13]. To recommend Seattle-PAP over the current standard of care (bubble nCPAP), a larger comparative trial is needed. Thus, the present study is designed to compare the incidence of respiratory failure over the first weeks of life between the two groups. While the present study is not designed to determine differences in longer-term clinical outcomes (BPD), prespecified secondary analyses are planned.

We acknowledge a number of challenges in the design of the trial. First, the potential benefits and risks of Seattle-PAP versus conventional bubble nCPAP (FP-CPAP) may not be uniform across infants born at all GAs. To that end, we determined a priori to perform a subgroup analysis on infants born after <27 weeks versus those born after 27 to 30 weeks of gestation. Second, our recruitment window provides enrollment up to 72 h of life, irrespective of previous respiratory support (mechanical ventilation or CPAP) and likely over a wide range of acutely impaired lung function. We recognize that this potential heterogeneity increases the risk of type II errors [36]. Third, some infants allocated to Seattle-PAP may receive a brief period of FP-CPAP prior to randomization, which conceivably could affect the interpretation of the results. We attempted to restrict the impact of this by excluding from the trial infants who have received more than 72 h of FP-CPAP, which we felt to be the shortest window in which seeking parental consent would be feasible. Finally, blinding of the interventions, which would have been preferable from a study design perspective, was not feasible, due to the complexity of building and operating a system that disguised which device was providing respiratory support to the infant, yet allowed the clinical care team to monitor effective engagement of the infant with the pressure-generating system.

The results of the present study will inform the design of larger multicenter trials that investigate more fully the benefits and risks of bubble nCPAP in low- and middle-income countries. Since CPAP failure in developing countries may result in neonatal death, potential use in these countries would be even more attractive [11, 14]. While previous investigators have described barriers to successful use of bubble CPAP in such settings (fixation devices are bulky and cover much of the infant’s face; interference with parental interaction and feeding; trauma to the nasal skin or septum; and need for nursing vigilance to ensure an adequate seal), others have shown that bubble nCPAP can be applied effectively in resource-limited situations [14, 37, 38].

The airway pressure oscillations generated with Seattle-PAP, including the lower frequency of pressure oscillations, are modestly different from those generated with conventional bubble nCPAP [17, 18]. Although preclinical and small clinical studies conducted to date have not indicated there is a greater risk of adverse events associated with Seattle-PAP than with conventional bubble nCPAP [13, 17, 18], the present trial will provide an opportunity to observe any unanticipated SAEs.

Use of CPAP among preterm infants is associated with reduced hospital stay [16] and with savings exceeding $10,000 for every six neonates treated with CPAP [39]. Given the interrelatedness of health, availability of resources, and the constraints on health-care budgets, as well as the substantial resource utilization by preterm neonates, improving health-care status is likely to have important cost implications; thus, we will perform a formal economic evaluation ancillary to the proposed RCT with the goal of informing the practice of effective and efficient health care.

### Potential impact

Seattle-PAP is a promising new bubble CPAP delivery system that may reduce the need for tracheal intubation and mechanical ventilation among preterm neonates. The Seattle-PAP trial will provide relevant and timely evidence on the efficacy and safety of the Seattle-PAP device compared to conventional bubble CPAP (FP-CPAP) in this vulnerable subgroup of patients.

## Additional files

Additional file 1: Standard Protocol Items: Recommendations for Interventional Trials (SPIRIT) 2013 Checklist: recommended items to address in a clinical trial protocol and related documents. (DOC 120 kb)

Additional file 2: **Figure S1.** NCH-NRN ventilation weaning protocol. (JPG 1579 kb)

Additional file 3: **Table S1**. Baseline characteristics and secondary outcomes of the Seattle-PAP trial [40–45]. (DOCX 21 kb)

Additional file 4: **Table S2.** Additional data collected during the study period. (DOC 43 kb)
