# Supplementary figures and images for: Evaluating the efficacy of Seattle-PAP for the respiratory support of premature neonates: study protocol for a randomized controlled trial
Source: Trials. 2019 Jan 18;20:63. doi: 10.1186/s13063-018-3166-6 (PMC6339409; doi:10.1186/s13063-018-3166-6)

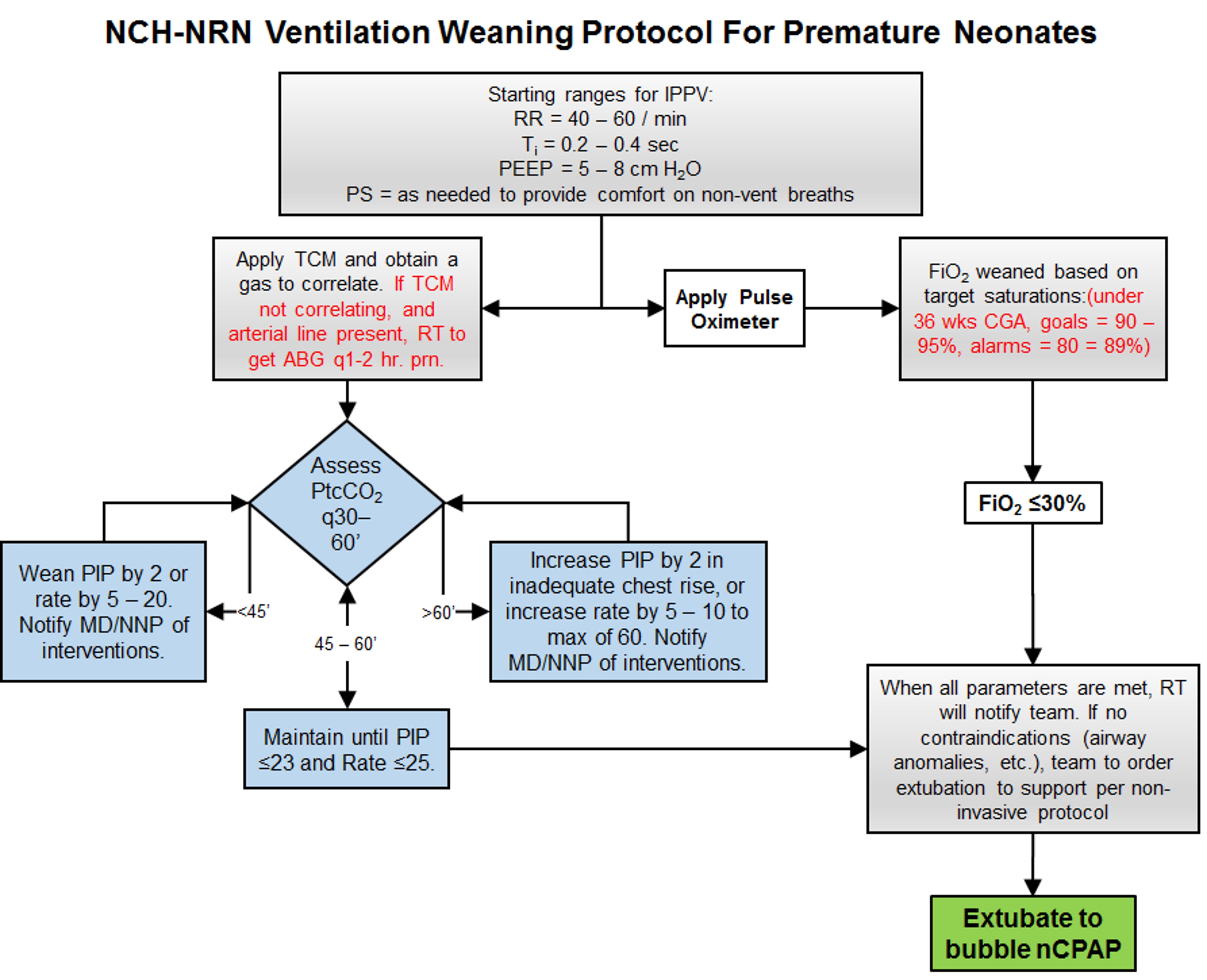

Supplement: Supplementary file 2 — Figure S1. NCH-NRN ventilation weaning protocol. (JPG 1579 kb) [file 13063_2018_3166_MOESM2_ESM.jpg]
