# Supplementary material for: Evaluating the efficacy of Seattle-PAP for the respiratory support of premature neonates: study protocol for a randomized controlled trial
Source: Trials. 2019 Jan 18;20:63. doi: 10.1186/s13063-018-3166-6 (PMC6339409; doi:10.1186/s13063-018-3166-6)
Supplement: Supplementary file 3 — Table S1. Baseline characteristics and secondary outcomes of the Seattle-PAP trial [40–45]. (DOCX 21 kb) [file 13063_2018_3166_MOESM3_ESM.docx]

**Additional file 2: Table S1; Secondary outcomes**

| Secondary outcomes |
| --- |
| - Individual components of primary outcome - All-cause mortality at 36 weeks gestational age. - All-cause mortality before first discharge home. - Length of hospital stay prior to discharge home, including days at transfer hospital (if applicable). - Bronchopulmonary dysplasia assessed at 36 weeks gestational age.(40) - Use of postnatal steroids for treatment of BPD - Need for re-intubation by birth weight strata (< 750g; 750g – 999g) beyond 72 hours. - Total duration of positive pressure respiratory support (e.g. mechanical ventilation plus either Seattle PAP or FP-CPAP) up to the time of discharge from the NICU. - Total time on supplemental oxygen until discharge. - Pulmonary air leaks identified radiologically by a masked pediatric radiologist. - Nasal deformities, as defined by Robinson et al.(41) - Intestinal perforation diagnosed by free gas in the peritoneal cavity on abdominal radiograph or at laparotomy. - Necrotising enterocolitis, diagnosed at surgery, autopsy or by the radiographic findings of pneumatosis intestinalis or hepatobiliary gas (Bell stage II).(42, 43) - Time to establish full feeds (no longer requiring parenteral nutrition). - Weight gain and feeding performance, including weight gain from birth to hospital discharge, proportion of infants fully breastfed at discharge, number of days receiving any intravenous fluids, number of days to reach full suck feeds (defined as tolerating suck feeds without any requirement for intravenous fluids or nasogastric/orogastric feeds for >24 hours) and time to establish full feeds (no longer requiring parenteral nutrition). - Nosocomial infections, defined as positive blood culture, positive CSF culture and/or diagnosis of pneumonia. - Intraventricular haemorrhage grade III-IV and/or periventricular leukomalacia and/or ventriculomegaly on cranial ultrasound.(44) - Retinopathy of prematurity at routine ophthamological examination beginning at 32 weeks gestational age; graded according to the international classification, as stage 3 (fibrovascular proliferation), stage 4 (partial retinal detachment) and stage 5 (total retinal detachment).(45) - Cost: estimated differences between the interventions based on the costs of equipment, care in NICUs, costs associated with hospital stay. |
