# Supplementary material for: Evaluating the efficacy of Seattle-PAP for the respiratory support of premature neonates: study protocol for a randomized controlled trial
Source: Trials. 2019 Jan 18;20:63. doi: 10.1186/s13063-018-3166-6 (PMC6339409; doi:10.1186/s13063-018-3166-6)
Supplement: Supplementary file 4 — Table S2. Additional data collected during the study period. (DOC 43 kb) [file 13063_2018_3166_MOESM4_ESM.doc]

**Additional file 3: Table S2**: Additional data collected during study period

| **Data Collection Topic** | **Specific Data Elements** | **Time of Data Element** |
| --- | --- | --- |
| **Maternal/Infant Demographics Form** | *Maternal:* maternal age; gravida/parida status; maternal ethnicity; maternal education level; maternal marital status; maternal prenatal care status; maternal pregnancy and delivery complications (e.g. - chorioamnionitis; preterm prolonged rupture of membranes, placental abruption, etc.); smoker; substance abuse during pregnancy  *Infant:* gestational age; date and time of birth; delivery hospital; birth weight, length, and head circumference; ethnicity | Completed within one week of enrollment |
| **Screening Checklist Form** | Meets inclusion criteria and none of exclusion criteria; date of randomization | As soon as possible following delivery |
| **Enrollment and Randomization Form** | Date and time of randomization; treatment allocation; date and time of transition to intervention;iInitial CPAP settings at enrollment | As soon as possible following treatment allocation |
| **Post-Delivery Clinical Information Form** | *In-utero* screening and findings; maternal and fetal health concerns; tocolysis; antenatal steroids, magnesium sulfate and other maternal medications; mode of delivery; APGAR scores; multiple gestation; NICU admission temperature; admission diagnosis / diagnoses; cord pH; infant admission pH; delayed cord clamping and time of delay; delivery room management (e.g. – intubation, chest compressions, surfactant administration, etc.); ventilation method used in delivery room; highest % oxygen requirement required immediately following delivery; oxygen level weaned to prior to leaving delivery room | Within 1 week of enrollment |
| **Transfer Form** | ***Employed if infant requires transfer to Nationwide Children’s Hospital main campus***  Date of transfer; reason for transfer | Within 72 hrs of transfer (if transferred) / At discharge (if not transferred) |
| **Respiratory Status Form** | *First 7 days of life, hourly data collection; 3 times daily thereafter*  Date; day of life; ventilation support type; intubation status; extubation status, date, time, and justification (if applicable); FiO_2_ settings; oxygen saturation level; highest FiO_2_ setting required; change(s) in respiratory support settings | Ongoing until 36 weeks PMA or discharge, whichever comes first |
| **Medication Form (*Daily*)** | Medications, dosages, and frequency of use during hospital stay | Ongoing until 36 weeks PMA or discharge, whichever comes first |
| **Length, Weight, and Feeds Form (*Daily*)** | Weight; length (if recorded); enteral feeding intake; feed type | Ongoing until 36 weeks PMA or discharge, whichever comes first |
| **Blood Transfusion Form (*If Needed / As Needed*)** | Date of transfusion; type of transfusion (e.g. – packed red blood cells, platelets; whole blood; etc.); volume | As needed |
| **Echocardiogram (ECHO) Results Form** | Date of ECHO; presence of patent ductus arteriosus (PDA); presence of other non-exclusive cardiac shunt (patent *foramen ovale* / atrial septal defect); ECHO findings (e.g. – atrial and/or ventricular hypertrophy, evidence of pulmonary hypertension, etc.); use of medications for PDA closure; need for surgical PDA closure and date of surgical closure (if applicable) | As needed |
| **Complications / Adverse Events Form** | Study participants are monitored for the following complications and/or adverse events, severity and date of diagnosis (if applicable): pulmonary interstitial emphysema (PIE); pneumothorax; pulmonary hemorrhage; bronchopulmonary dysplasia (BPD); pulmonary hypertension; intraventricular hemorrhage (IVH); retinopathy of prematurity (ROP); periventricular leucomalacia (PVL); necrotizing enterocolitis (NEC); positive microbial cultures (sepsis); need for intubation >72 hours of life; nasal pressure lesions; esophageal perforation; ventilatory support device failure or malfunction; expiration / death | 1) Day of life 28  2) Study exit |
| **Study Exit Form** | Date of study exit; study disposition (withdrawn from study; reached 36 weeks PMA); treatment success or failure; reason for failure (if applicable); head circumference; date of transition off of CPAP; corrected gestational age at time of transition from CPAP; total number of days on CPAP; medications at time of CPAP transition | 36 Weeks PMA |
| **Discharge Status Form** | Discharge / disposition date; disposition type (expiration, discharge home, discharge to other facility); use of oxygen therapy and settings at time of discharge; date to reach full enteral feeds; morbidities during hospitalization; total number of surfactant doses given during hospital stay | Within 1 week of hospital discharge |
| **Additional Data Form** | Additional data regarding ventilatory support requirements after study end; other morbidity detail occurring after study end and before hospital discharge | Within 1 week of hospital discharge |
